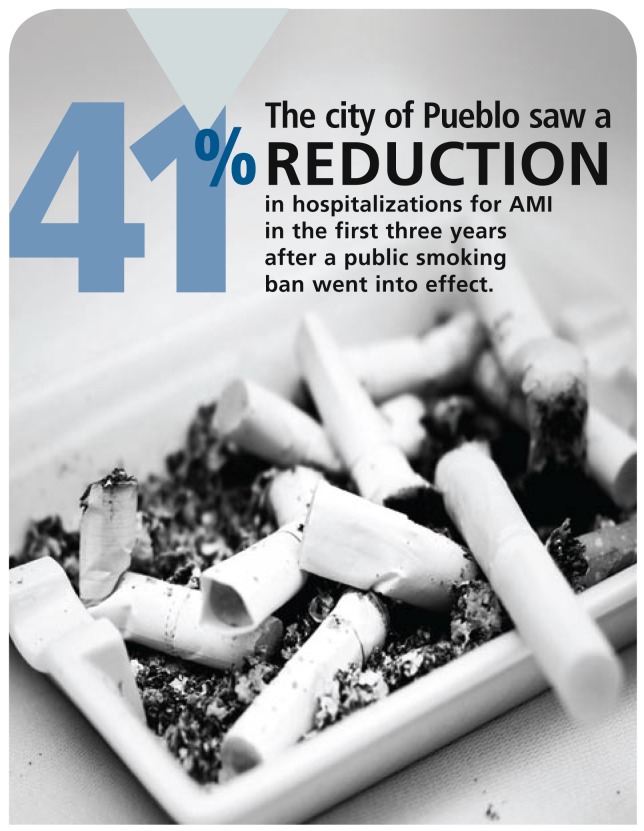# POLICY: Smoking Bans Yield Long-Term Benefits

**Published:** 2009-03

**Authors:** Adrian Burton

When it comes to linking cigarette smoke to heart attacks, one might assume that such a link would apply only as long as one is smoking or being exposed to secondhand smoke. That’s not necessarily true, however, at least according to a follow-up study of people living in and around Pueblo, Colorado. In the 2 January 2009 issue of *Morbidity and Mortality Weekly Report* (*MMWR*), researchers reported that hospitalizations in Pueblo for acute myocardial infarction (AMI) showed a steady, continuous decline three years after the city introduced a smoking ban in all public buildings.

In November 2005, researchers first began to report that a smoking ban in all public buildings in Pueblo was associated with a significant reduction in the number of hospitalizations for AMI [see *EHP* 114:A154 (2006)]. In the 18 months after the ban came into force, the number of such hospitalizations fell 27%, from 257 to 187 per 100,000 person-years. Only a small reduction was seen for the population living outside the city limits, and no reduction was seen in neighboring El Paso County—areas unaffected by the ban.

The new figures published in *MMWR* examine the three years since the smoking ban began and show that the number of hospitalizations for AMI has continued to decline to 152 per 100,000 person-years, says Robert Alsever, medical director for health information at the Parkview Medical Center in Pueblo and first author of the update report. That’s a 19% reduction over the figure for the first 18 months after the ban began and a total 41% reduction since the ban started, he says. The point at which these improvements will level off remains to be seen.

In a review published in the February 2009 issue of *Preventive Medicine*, Lorenzo Richiardi and colleagues noted that at least 10 studies in the United States and Europe had reported a reduction in the number of hospital admissions for AMI in the months following the introduction of regulations banning smoking in public places. The decreases observed ranged from 8% in New York State to 40% in Helena, Montana. Specifically, the Helena study found a 40% decline in hospital admissions for AMI during a 6-month smoking ordinance within Helena city limits; however, this was a relatively small population study.

An editorial note that accompanies the *MMWR* report observes that a meta-analysis of seven other studies and one unpublished study yielded a pooled estimate of a 19% reduction in AMI hospitalization rates after implementation of public smoking bans. Three studies suggested these reductions were more pronounced among non smokers than among smokers.

The Pueblo study, however, did not assess what portion of the observed decrease in hospitalizations could be attributed to reduced secondhand smoke exposure among nonsmokers or to quitting among smokers. Even though the prevalence of smoking dropped in Pueblo County as a whole, the difference over time was not statistically significant. As the editorial note explains, “the ecologic nature of this study precludes definite conclusions about the extent to which the observed decline in AMI hospitalizations in the city of Pueblo was attributable to the smoke-free ordinance.”

One concept that may begin making its way into tobacco control policies and programs is that of “thirdhand smoke,” which refers to the chemicals in cigarette smoke that settle on the surfaces inside a home. Jonathan Winickoff, a pediatrician at the Dana-Farber/Harvard Cancer Center, is one of a growing number who believe these deposited chemicals pose a hazard to occupants’ health days after a cigarette is stubbed out. “Children are particularly likely to come into contact with this third-hand smoke,’” explains Winickoff. “The chemicals in smoke may stick to objects for many days and are gradually released back into the air, so infants and children breathe in and ingest tobacco toxins as they crawl over, mouth, and play on contaminated surfaces.”

In a survey published in the January 2009 issue of *Pediatrics*, Winickoff’s team asked U.S. adults to describe their beliefs about the health effects of second- and thirdhand cigarette smoke. Many people reported believing that thirdhand smoke is far less of a health concern for children than secondhand smoke. Those who did report believing thirdhand smoke poses a hazard to children were more likely to enact strict smoking bans inside their homes. Says Winickoff, “These findings suggest that if more people understood the dangers of thirdhand smoking, especially to their children, we might see more nonsmoking policies adopted in the home.”

## Figures and Tables

**Figure:**